# Four Interesting Cases at Cloverdale Stock Farm

**Published:** 1897-11

**Authors:** J. Curtis Michener

**Affiliations:** Colmar, Pa.


### FOUR INTERESTING CASES AT CLOVERDELL STOCK FARM.[1]

By J. Curtis Michener, V.S.,
COLMAR, PA.

Case I. *Acute dyspnœa in a colt.*—During the month of January the past season's colts were having strangles. They were under the treatment of Dr. Seaman, the resident veterinarian. During the day and evening of the 29th, one of the sucking colts was noticed to be discharging very freely, though otherwise quite well. As night came on we had a very piercing, cold wind, the worst of the season; the stable had many large cracks, being almost as cold as out of doors. The watchman, at midnight, found the colt roaring until it could be heard above the wind several yards away. The doctor, on being called, sent for me in great haste, with instructions to bring along my tracheotomy tube. I found the colt very cold, mouth wide open, staggering about, shaking its head, and making the same kind of a noise that a horse makes if you hold his nostrils shut for several minutes. The doctor demanded that I operate without a moment's delay, or it would be too late. I told him I would not do it either then or there. We first carried the colt into a warm stable by the engine-room, wrapped

[1] Presented for consideration at the semi-annual meeting of the Pennsylvania State Veterinary Medical Association, Franklin, Pa., September, 1897.

him in warm blankets, put his nose over steaming water, and fomented the nose and throat with warm water.

The doctor, becoming more nervous every minute, said, if I would not operate, he would go for my son, Mayhew. I told him to not be foolish, as the colt would be relieved or die before he could be summoned; when, lo and behold! the colt gave a spasmodic snort, and blew out great plugs of hard pus, looked around a moment in relieved amazement, then walked over to its dam, and took a hearty suck. It was entirely relieved, and went right on making a rapid and complete recovery. We called it a case of being frozen up.

CASE II. *Paraphimosis in a stallion.*—On April 1st I was called to attend the celebrated old stallion "Red Wilkes," and found him with the end of the penis swollen to the size of a man's head, strangulated, and hourly growing worse; a case of paraphimosis. It gave the horse much uneasiness; he stamped about, switched his tail, and his countenance bespoke much anxiety. First, I made six punctures, from which serum dropped freely. Next, I fomented with warm water for several minutes, when the stricture relaxed so far as to allow the hand to be passed into the sheath, when the cause of the trouble was found. Whenever the plank flooring became wet, it was sprinkled with sharp white sand to prevent slipping. A large quantity of this had adhered to the penis and been carried back into the sheath, starting a violent irritation.

After a thorough cleansing and anointing with cosmoline, the penis was readily returned, but would soon drop out about eight inches. Plenty of exercise out in the cool air soon caused the parts to rapidly resume their normal size and position. In three days he was all right and serving mares.

CASE III. *Dislocation of the coxo-femoral articulation; reduction.*—This occurred on April 22d. An old mare, running with others at pasture, was found holding a hind-foot up about eighteen inches, and turned inward on a line with the other foot, keeping in constant motion, evincing great pain. When I arrived, she was down, resting well. I examined the foot for injury, and the bones for fracture, but was unable to make a diagnosis. I drove her up, when the case became very plain. She held the foot up forward and under the belly. It could not be forced to the ground, and the head of the femur could be plainly felt above and behind the acetabulum. I placed a strap around the pastern with a rope attached in the hands of four men. I had one man hold her by the bit and mane, and another one to brace up the opposite hip, then directed the men at the rope to pull strong and steady forward and outward, while I clasped the hip with one hand and pushed the protruding bone with the palm of the other. It went to its place without difficulty with a snap plainly heard. She put her foot down and walked off without lameness. The next day she was trotting about as though nothing had happened.

CASE IV. *Ulceration and perforation of the intestine from eating foreign products.*—August 9th, a sucking colt was found to be drooping and weak. When called I found it in a state of collapse, with abdomen swollen and tender, but nothing like colicky pains had been noticed. It died in four hours without a struggle. On opening the peritoneal cavity, a quantity of bloody serum, a little fecal matter, and about twelve worms, ascarides (*Ascaris magalocephala*) made their escape. There was general peritoneal inflammation. I found the floating end of the cæcum mortified and having a ragged opening about an inch in diameter, through which the worms and fecal matter had made their escape. Just inside the rent was found a hard, tough ball, which was composed of hard, woody threads. I examined the pasture and found the colt had reached between the slats of tree-frames and stripped the bark from off young maples. For the sake of being fashionable, I called it a case of appendicitis.

